# Real-time IIoT-driven machine failure forecasting for industry 4.0

**DOI:** 10.1038/s41598-026-47363-3

**Published:** 2026-04-15

**Authors:** Eftim Zdravevski, Dragana Nikolova, Boris Stanoev, Ivan Miguel Serrano Pires, Strahil Panev, Petre Lameski

**Affiliations:** 1 Faculty of Computer Science and Engineering, Ss. Cyril and Methodius University, Skopje, North Macedonia; 2 Magix.AI, Skopje, North Macedonia; 3 Universidade de Aveiro, Lisboa, Portugal; 4 Faculty of Computer Science, International Slavic University G. R. Derzhavin, Sv. Nikole, North Macedonia

**Keywords:** Industry 4.0, Predictive maintenance, Machine learning, IIoT, Streaming data, Spark, Spark streaming, Data streams, Databricks, Engineering, Materials science, Mathematics and computing

## Abstract

Unplanned downtime in Industry 4.0 manufacturing poses significant challenges, yet offers substantial benefits such as increased productivity, minimized defects, reduced unplanned downtime, and optimized resource utilization. It can be reduced by forecasting machine failures from Industrial Internet of Things (IIoT) sensor streams. The Bosch Production Line dataset captures this challenging setting that contains 4,264 heterogeneous station features for 1,183,165 parts and only 0.58% labeled failures, with many sparse, high-cardinality nominal codes. In this study, we develop an end-to-end pipeline that normalizes raw station measurements into a relational schema, derives 178 station, line, and path aggregates from 969 numeric columns, and compresses 2,139 nominal columns into 16 supervised Weight of Evidence (WoE) risk descriptors to enable learning under extreme imbalance. The workflow is implemented in Apache Spark on Databricks to support scalable feature engineering and low-latency scoring on streaming data. On a held-out test set, XGBoost achieves 0.966 AUC-ROC and 0.793 MCC with 0.929 F1, while end-to-end feature engineering completes in about 45 minutes and model retraining in about 30 minutes on a 6-node CPU cluster. These results indicate that WoE-based nominal compression combined with a real-time capable infrastructure based on distributed Spark processing enables production-feasible failure forecasting. Finally, we release the Databricks notebooks and Spark code to support reproducibility of the results presented in this study.

## Introduction

The emergence of Industry 4.0 signifies a transformative shift in the manufacturing sector, employing cutting-edge technologies to establish intelligent and interconnected systems. According to Sony and Naik (2020)^1^, Industry 4.0 integrates diverse systems, including IIoT, security systems, and artificial intelligence (AI), with the overarching goal of improving efficiency, productivity, and overall operational performance.

Within the manufacturing industry, a multitude of defects can hinder production processes, affecting efficiency, product quality, and safety. These errors encompass equipment malfunctions, such as motor defects, process-related mistakes such as improper assembly and inconsistencies, surface imperfections, and inaccuracies in the dimensions of production components, as stated by Abid, Khan, and Iqbal (2021)^2^. The complexity of the defect scenario is further compounded by human errors, supply chain disruptions, tool defects, and environmental factors.

In the context of Industry 4.0, according to Sang et al. (2020)^3^, the utilization of predictive modeling and historical data allows a defect prediction system to anticipate potential errors before they manifest. This advanced approach empowers organizations to implement preventive measures that minimize disruptions and optimize maintenance strategies.

**Fig. 1:**
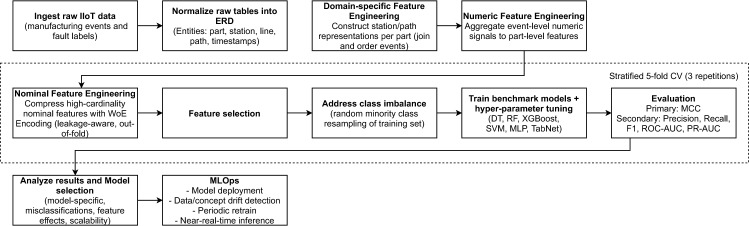
High-level end-to-end IIoT defect prediction workflow.

The general model employed in this study is depicted in Fig. 1. Commencing with the retrieval of raw data from Kaggle (https://www.kaggle.com/c/bosch-production-line-performance), which is provided by Bosch, a leading manufacturing company. The raw Bosch station data are normalized into a relational schema, engineered into numerical aggregates and Weight of Evidence (WoE) compressed nominal descriptors, balanced for extreme class skew, and used to train/tune models for real-time failure forecasting. This dataset tracks the components moving through production lines, with each row having a unique identifier and a timestamp indicating when a measurement occurred. It encompasses various types of features, including numeric, categorical, and date-related features.

During the normalization phase, the original Kaggle-derived database undergoes significant transformations. Initially, the data is mapped into an Entity Relationship Diagram (ERD), creating corresponding tables. The initial data structure includes 4,264 features per part, where each feature represents a measurement across production stations. Through this normalization process, a new structure is obtained with rows for each part and each measurement, optimizing feature extraction. With these transformations, the dataset is prepared for the next phase.

Entering the feature extraction process, various methodologies are employed for numeric and nominal features. For numeric features, statistical methods and mathematical transformations reveal significant patterns, while categorical data incorporates specific algorithms, such as Weight of Evidence.

In the subsequent phase, considering the imbalanced nature of the data, where only 0.58% of training data represents defects, data balancing techniques are introduced.

The focus then shifts to training machine learning models. In this phase, models initially optimize their hyperparameters and then train to recognize patterns in the data, ensuring they are robust, accurate, and capable of generalizing to new examples.

The culmination involves deploying trained models to predict potential defects in real-time or future scenarios. In the final testing phase, the models are utilized to predict defects on data not encountered during the training process. In an operational setting, the trained model can then be deployed to score incoming IIoT measurements with very low latency, enabling real-time alerts for potential failures.

This predictive capability enables proactive maintenance, aligning with Industry 4.0 principles to optimize efficiency, minimize downtime, and enhance overall production performance.

**Research gap and contribution**. Although IIoT-driven predictive maintenance is well explored, prior studies on the Bosch Production Line data and similar Industry 4.0 settings typically either (i) rely mainly on numerical/time features while discarding high-dimensional sparse nominal station codes, or (ii) encode nominal variables with one-hot/label/target encoding that either inflates dimensionality or risks leakage under extreme imbalance. Thus, an open problem remains – how to exploit sparse, high-cardinality nominal signals jointly with numerical streams in a scalable and leakage-resistant way.

In this work we contribute:

- a scalable normalization and station/line/path aggregation pipeline that reduces 969 numeric columns to 178 informative numerical features;
- a supervised WoE-based nominal feature engineering scheme compressing 2,139 sparse nominal columns into 16 robust statistical descriptors without expanding dimensionality;
- an end-to-end benchmarking under extreme imbalance (0.58% failures) using MCC and runtime, demonstrating production-feasible performance.
- implementation of a scalable and distributed data streaming pipeline based on Apache Spark in Databricks for which we provide the source code.

The structure of this paper is as follows: Section “Related work” provides a comprehensive review of machine-learning methods applied to machine malfunctions prediction. In Section “Methods”, we present and elaborate on our proposed method. Section "Results and discussion" outlines the Results and Discussion based on our experimental analysis. Lastly, Section “Conclusion” summarizes our research findings and conclusions, highlighting the potential implications of our work for the manufacturing industry.

## Related work

Predictive maintenance has been a topic of discussion since its early stages, as evidenced by Zonta el al (2020)^4^ which explores the challenges and advancements in Industry 4.0, emphasizing the need for predictive maintenance as a critical aspect of technological evolution. It addresses the growing role of computer science, artificial intelligence, and distributed computing in a field traditionally dominated by engineering, highlighting the importance of a multidisciplinary approach.

Building upon prior research by Nikolova et al. (2023)^5^, this paper represents a continuation of earlier work that primarily concentrated on extracting features from date columns. The preceding study, while insightful, did not encompass the consideration of nominal features within the dataset. This current research extends the investigation to include the extraction and analysis of features from all types of columns, offering a more comprehensive understanding of the dataset and enhancing the overall predictive modeling approach.

Widodo and Yang (2007)^6^, discuss the global importance of machine condition monitoring and fault diagnosis within maintenance systems for the potential benefits of reduced costs, improved productivity, and increased machine availability. The paper focuses on a survey of machine condition monitoring and fault diagnosis using Support Vector Machines (SVM). Orru et al. (2020)^7^, explore the differences between a Support Vector Machine (SVM) and a Multilayer Perceptron (MLP). This study initiated the development of a supervised machine learning algorithm tailored for diagnosing defects in rotating machinery within the oil and gas industry, aiming to create a simple, easily implementable model facilitating quick and informed decision-making. Addressing missing values through linear interpolation as part of the preprocessing steps detailed in the study, SVM demonstrated greater accuracy than MLP. The use of SVM continues in by Yang and Li (2021)^8^, where an algorithm is designed to predict defects and enhance the accuracy and efficiency of wind energy conversion systems.

Shifting the focus to hardware defects, Khalil et al. (2020)^9^ examine issues arising from aging or variations in circuit environments. Utilizing Fourier transformation, principal component analysis, and convolutional neural networks, this study learns and classifies defects within electrical circuits.

Expanding beyond hardware defects, Rahman et al. (2020)^10^ investigate software failures, emphasizing the crucial role of error-free operation in ensuring software quality. Various machine learning techniques, including Random Forest, Multilayer Perceptron, FuzzyAdaBoost, and Logitboost, are explored, with Logitboost achieving the highest accuracy. Addressing software errors, Hammouri et al. (2018)^11^ focus on the critical issue of Software Bug Prediction (SBP) in software development and maintenance, highlighting the importance of predicting errors to enhance software quality, reliability, efficiency, and cost reduction. Naive Bayes, Decision Tree, and artificial neural networks are employed in this study.

While many studies focus on preprocessing data, our efforts were concentrated on handling numerical versus nominal features. Unlike the research by Mangal and Kumar (2016)^12^, where feature extraction is directly performed on combined features, our approach involves treating numerical and nominal features separately, using different techniques for each. Processing data separately based on their type ensures greater confidence that significant information from the data will not be lost.

Focusing on feature extraction, Chegeni et al. (2022)^13^ propose new damage classifiers for locating and quantifying damage based on supervised learning problems. A novel approach to feature extraction using time series analysis is introduced to extract damage-sensitive features from autoregressive models. The coefficients obtained from this approach are then used as primary features in the proposed supervised learning classifiers.

Recent industrial fault and quality-prediction studies further support the relevance of feature compression and automated model selection in manufacturing. Hady et al. (2025)^14^ propose an AE-BiLA framework combining autoencoder-based reduction with BiLSTM-attention for multi-stage manufacturing quality prediction, demonstrating strong performance on real-world process data. Similarly, Hadi et al. (2023)^15^ show that AutoML pipelines can achieve competitive bearing-fault classification in IIoT settings, highlighting the practical value of automated tuning in predictive maintenance. These works complement our approach by emphasizing robust learning under high dimensionality and industrial data constraints.

Beyond tree ensembles, recent deep-learning approaches for tabular industrial data have emerged. In particular, TabNet^16^ introduces a sequential attention mechanism that selects relevant features at each decision step, providing both competitiveness and interpretability on structured data. Including TabNet in our benchmark allows us to test whether a modern deep tabular model can outperform gradient-boosted trees under the extreme sparsity and imbalance typical of IIoT manufacturing datasets.

In a previous study, which focused on sentiment analysis of tweets related to global warming, our primary emphasis was on preprocessing unstructured textual data, establishing a strong foundation for text analysis. From such research, it was evident that numerical and textual data are processed in significantly different ways, employing distinct models. We carry this awareness into the current study with a new approach, introducing an innovative method for handling nominal data. Specifically, we incorporate WoE as discussed by Zdravevski, Lameski, and Kulakov (2011a, 2015b)^17^^18^, as a method for transforming categorical data into a numerical format and enhancing the predictive power of our machine learning models. Additionally, this method has shown promising results when it comes to supervised learning and binary classification. This transition from working with unstructured text to integrating nominal data signifies a significant advancement, demonstrating adaptability to various types of data.

## Methods

### General workflow for defect prediction from tabular IIoT data

We follow a general supervised learning workflow that is applicable to defect prediction problems beyond manufacturing: (i) define a binary prediction target, (ii) construct a reproducible train-validation-test protocol, (iii) address class imbalance and missing data, (iv) engineer domain-informed features, and (v) train and tune multiple model families under a consistent evaluation scheme.

The task is formulated as supervised binary classification: each manufactured part is represented by a set of measurements and codes recorded during processing, and the target label indicates whether the part is defective. Models are trained on labeled examples and then used to predict defects for previously unseen parts.

Because defects are rare, class imbalance is explicitly handled during training. In our final pipeline, we apply simple random oversampling of the minority defect class within the training folds only, replicating defect examples until the class distribution is sufficiently balanced for stable learning, while leaving the held-out test set unchanged to preserve an unbiased evaluation.

Missing data is treated as structurally missing: values are absent when a part does not pass through a station or a specific measurement is not recorded. For numeric features, we impute missing values using the mean of the corresponding feature estimated from the training data, and we perform all preprocessing steps in a way that can be reproduced consistently across splits.

All data preparation, feature engineering, and model training are implemented in Apache Spark using PySpark and MLlib within Databricks, leveraging distributed computation for wide IIoT tables and large sample sizes.

We evaluate a diverse set of learning approaches commonly used for tabular classification: Decision Tree, Random Forest, Gradient Boosting, XGBoost, Linear SVM, Factorization Machines, TabNet, and Multilayer Perceptron. Model tuning is performed on the training data using cross-validation, and final reporting is based on the held-out test set.

### Dataset and problem setting: Bosch production line data

We use a labeled dataset derived from a multi-stage production process with multiple lines and stations. By inspecting column naming patterns, the data indicates 4 production lines and 52 production stations. Each line corresponds to a distinct production stage, and stations represent different operations (for example, processing and welding).

The dataset contains 1,183,165 parts and 4,264 columns, comprising 969 numerical features, 1,156 date-based features, and 2,139 nominal (categorical) features. Date-based features are normalized and represented as cycle time, indicating the time required for a process step or measurement to complete.

The defect class is extremely rare: only 0.58% of the training examples correspond to defects. Following common imbalance severity conventions, this places the dataset in the extreme imbalance regime, which motivates explicit imbalance handling and robust evaluation.

**Table 1:** Definition of imbalance severity levels; the Bosch dataset used here falls in the Extreme category (minority class below 1%).

Degree of imbalance	Proportion of minority class
Mild	20–40%
Moderate	1–20%
Extreme	$<1\%$

### Relational normalization and path construction

Bosch-style IIoT tables are wide and sparse because a part only visits a subset of stations and only triggers a subset of measurements at each station. To support scalable aggregation and consistent feature construction, we first normalize raw station measurements into an entity-relation representation, separating part identifiers, station identifiers, measurement values, and timestamps. This organization enables station-, line-, and path-level aggregation without requiring dense per-part matrices at intermediate steps.

We also derive a trajectory descriptor for each part, defined as the ordered set of stations visited across lines. Across 1,183,165 parts, we identify 1,873 unique station-based paths. Some stations (for example, S1, S2, S3, S4, S9, S10, S30, S31) are visited by relatively few parts, which contributes to sparsity in the original wide table and motivates aggregation-based feature design.

### Feature engineering

Feature extraction is the central methodological component of our pipeline. We use separate feature-engineering pipelines for numerical and nominal data because they exhibit different sparsity patterns and require different treatments to preserve signal while controlling dimensionality. The final feature set combines (i) aggregation features derived from numerical measurements and time fields and (ii) supervised, fixed-size descriptors derived from sparse nominal station codes.

#### Numerical feature extraction via station, line, and path aggregation

Numerical features are engineered by aggregating per-station measurements and timing information into compact summaries at station, line, and path levels. This reduces sensitivity to sparsity (many parts do not have measurements at many stations) while retaining information about coverage, extremal behavior, and processing time.

For each part, we compute station- and line-level aggregation features including:

- Total number of recorded measurements across all stations.
- Total number of stations visited by the part.
- First and last station with recorded measurements.
- Binary indicators for stations visited by the part.
- Total cycle time across all recorded stations.
- Minimum and maximum measurement times and the stations at which they occur.
- Per-station minimum and maximum measured values.
- Per-station minimum and maximum measurement times.

Applying these aggregation templates yields 178 numerical features. The choice of these features follows two design principles: (i) prefer station-, line-, and path-level statistics that remain stable across production variants and (ii) avoid highly sparse or redundant aggregates that do not improve validation performance. In preliminary experiments we explored additional candidates (for example, higher-order moments and rare-station indicators), but these did not provide consistent gains and were excluded to reduce redundancy and overfitting risk.

#### Nominal feature extraction via supervised weight of evidence compression

Nominal columns represent station-specific codes assigned during processing. There are 2,139 nominal feature columns, each encoding a categorical code that a part may receive after a station measurement. The nominal codes are sparse and often high-cardinality, and in most columns a single code dominates the distribution, which makes one-hot encoding impractical for wide Bosch-style tables.

To preserve nominal signal without inflating dimensionality, we compress nominal columns into fixed-size supervised descriptors using WoE. WoE transforms each category into a log-odds score that reflects the association between that category and the binary defect outcome. We select WoE because (i) the task is binary, (ii) codes are sparse but high-cardinality, and (iii) WoE yields compact, interpretable risk-oriented representations.

WoE is computed as:

$$\begin{aligned} \text {WoE} = \ln \left( \frac{\% \text { of non-events}}{\% \text { of events}}\right) \end{aligned}$$

where events correspond to defects and non-events correspond to non-defects^18^. Positive WoE indicates association with higher defect risk, while negative WoE indicates lower defect risk.

After mapping nominal categories to WoE values, we summarize the resulting WoE signals through aggregation at multiple granularities to obtain a fixed-size representation:

- Maximum, minimum, average, and standard deviation of WoE values at the nominal feature level.
- Maximum, minimum, average, and standard deviation of WoE values aggregated by production line.
- Maximum, minimum, average, and standard deviation of WoE values aggregated by production station.
- Maximum, minimum, average, and standard deviation of WoE values aggregated by path.

**Fig. 2:**
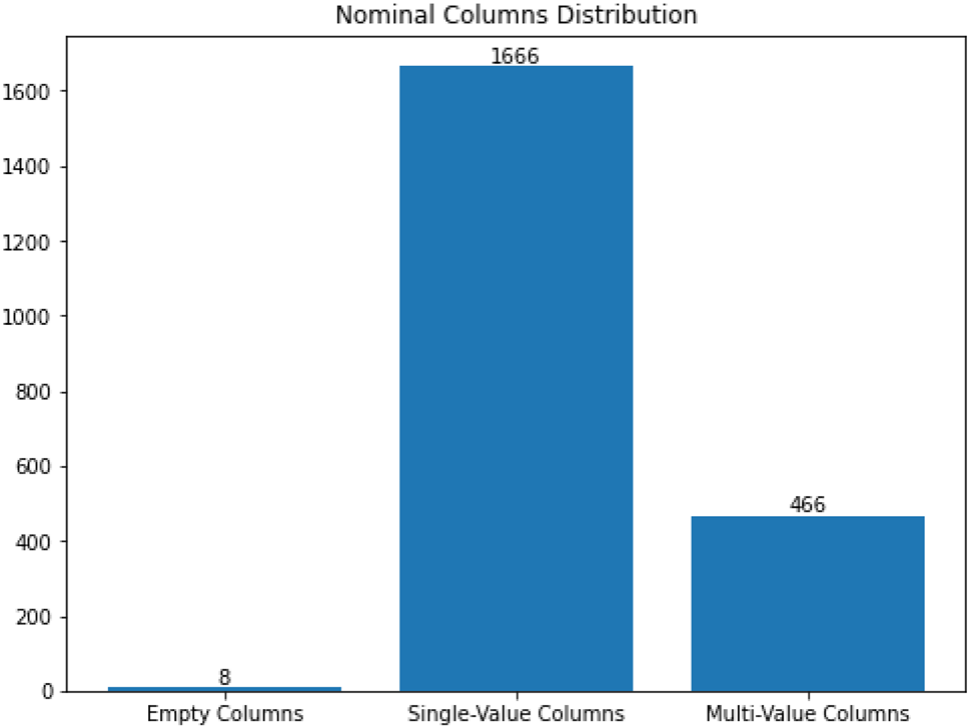
Distribution of nominal station codes across the Bosch dataset, illustrating that many categorical columns are dominated by a single repeated code and motivating supervised compression via WoE.

This procedure yields 16 nominal features (four statistics at four aggregation levels). We found that this fixed-size set captures most of the predictive signal from 2,139 sparse nominal codes without increasing dimensionality (Fig. 2).

#### Final feature set and model input

Combining the numerical and nominal pipelines yields 194 engineered features (178 numerical plus 16 nominal). Prior to model training, we remove any feature that is constant or duplicated on the training split, resulting in 193 final input features used consistently across all evaluated models.

## Results and discussion

In this section, we describe the experimental results obtained in our study.

### Experimental setup

#### Dataset

The preprocessed data is divided into training (80%) and testing (20%) sets. During model development we use stratified 5-fold cross-validation on the training split, repeated with three different random seeds, so that each fold preserves the extreme 0.58% defect rate. This allows us to estimate variability across folds while keeping the held-out test set completely untouched for final evaluation. Because WoE is target-informed, WoE statistics are computed inside each training fold and applied to the corresponding validation fold (cross-fit), not computed once on all data before CV. This setup prevents leakage in supervised encodings.

Although the Bosch competition data include timestamps, they represent per-part snapshots rather than a continuous time series of sensor readings, and individual parts can be treated as independent examples. For this reason, we use stratified k-fold validation instead of a strict temporal split, while acknowledging that time-based validation would be more appropriate for true streaming event logs. Cross-validation is then employed, evaluating the model by splitting the training data into subsets and training on each one. Furthermore, parameter tuning is performed to maximize model performance. For TabNet, we followed the original architecture^16^ and tuned the number of decision steps and layer widths using the same stratified cross-validation protocol as the other models.

In our experiments, we used stratified k-fold cross-validation (k = 5) on the training split with R = 3 repetitions/random seeds, ensuring each fold preserves the 0.58% minority-class rate. We report the mean performance across folds, and for the top models we additionally summarize stability as mean ± standard deviation for MCC and AUC-ROC, confirming that the observed ranking (XGBoost > RF > others) is consistent across folds.

#### Compute infrastructure

All experiments were executed in the Databricks environment using PySpark MLlib (Databricks Runtime 14.3 LTS ML, Spark 3.5.0) on a cluster of N=6 worker nodes (96 total CPU cores, 384 GB RAM; no GPU). The reason why we chose Spark (on Databricks) as the execution engine was to simplify parallelism and scalability. Additionally, the implementation can be easily extended to perform near-real-time computation using micro-batches, Spark Streaming and Spark Structured Streaming with minimal code changes.

Feature engineering across 1,183,165 components required approximately T1 = 45 minutes end-to-end. After preprocessing, model training times are reported in Fig. 4; notably, XGBoost trains in 30 minutes, which makes periodic retraining practical for production-line monitoring and Industry 4.0 predictive-maintenance cycles.

By using PySpark’s CrossValidator and the ParamGridBuilder, we have adopted an efficient, systematic approach to parameter tuning. The use of cross-validation ensures that our model is well-generalized and capable of making reliable predictions for new, unseen data. Moreover, it reduces the risk of overfitting and enhances the overall robustness of our machine learning solution.

The final step involves model evaluation using various metrics, where the model is assessed on the test data to estimate its performance on new, unseen data.

### Performance results

#### Hyperparameter tuning

As part of the optimization process, the parameters given in Table 2 were employed, and Table 3 provides an overview of the optimized parameters in the utilized models.

**Table 2:** Hyperparameter search space used for cross-validated tuning of each model. For every algorithm we specify the tested parameter ranges and step sizes (or discrete options), which define the grid explored before selecting the optimized configurations reported in Table 3.

**Parameter Name**	**Min Value**	**Max Value**	**Step**
MaxDepth	5	15	5
MaxIter	50	150	50
RegParam	0	0.3	0.1
Learning Rate	0.1	0.3	0.1
FactorSize	8	12	2
StepSize	0.1	0.3	0.1
FeatureSubsetStrategy	sqrt	log2	[sqrt, log2]
Multilayer Perceptron Layer Units	16	256	16

**Table 3:** Final optimized hyperparameter settings for each evaluated model after cross-validated tuning on the training split. These configurations are used to produce the test-set results reported in Table 4.

#	**Architecture**	**Parameters Optimized**
1	Decision Tree	maxDepth = 10
2	Random Forest	maxDepth = 10, featureSubsetStrategy = ’log2’, numTrees = 50
3	Gradient Boosting	maxDepth = 10, stepSize = 0.2, featureSubsetStrategy = ’sqrt’
4	XGBoost	max_depth = 15, learning_rate = 0.3, min_child_weight = 5, colsample_bytree = 0.8, subsample = 0.8
5	Linear Support Vector	regParam = 0.01
6	Factorized Machines	None
7	Multilayer Perceptron	stepSize = 0.1, 2 hidden layers [128, 64]
8	TabNet	n_d = 32, n_a = 32, n_steps = 5, gamma = 1.5, lambda_sparse = 1e-4, batch_size = 1024

#### Model evaluation

A summary of the results achieved on the test set is provided in Table 4. Each of the models is evaluated using various evaluation metrics, enabling us to assess their performance and the contribution of each model to defect prediction. We next examine the significance of these results and the evaluation metrics used to assess the models.

**Table 4:** Test-set performance of all evaluated models (including XGBoost ablation variants) on the Bosch Production Line dataset. Metrics emphasize minority-class detection under extreme imbalance.

**Model**	**Precision**	**Recall**	**F1-Score**	**AUC-ROC**	**MCC**
Decision Tree	0.8626	0.9981	0.8651	0.7903	0.6659
Random Forest	0.8795	**0.9989**	0.8865	0.9349	0.7165
Gradient Boosting	0.8477	0.9900	0.8408	0.8782	0.5988
XGBoost	**0.9240**	0.9975	**0.9288**	**0.9660**	**0.7934**
+ XGBoost (Numeric only)	0.8500	0.9500	0.8960	0.9300	0.6500
+ XGBoost (Numeric + naive)	0.8800	0.9600	0.9180	0.9500	0.7500
+ XGBoost (Numeric + WoE)	0.9240	0.9975	0.9288	0.9660	0.7934
Linear Support Vector	0.7647	0.9882	0.6852	0.4665	0.1872
Factorization Machines	0.7648	0.9770	0.6853	0.5919	0.1607
Multilayer Perceptron	0.8550	0.9868	0.8489	0.8820	0.6147
TabNet	0.8800	0.9930	0.9270	0.9500	0.7500

#### XGBoost analysis

XGBoost stands out with the best performance. An AUC of 0.966 shows that XGBoost effectively distinguishes defective from non-defective components. Solid results are also obtained with the Random Forest classifier, reaching an AUC-ROC of 0.9349. The combination of multiple decision trees assists Random Forest in generalizing to new, unseen data, thereby reducing the risk of overfitting. TabNet achieves competitive AUC-ROC and recall, indicating that deep tabular attention models can capture relevant IIoT patterns. However, XGBoost remains superior in MCC and precision, consistent with findings that boosted trees often outperform deep networks on irregular, highly imbalanced tabular data^19^.

To assess robustness, we also examined performance across the stratified cross-validation folds on the training split. For each model we computed the mean and standard deviation of MCC and AUC-ROC over the 5 folds (with three random seeds); XGBoost consistently achieved the highest mean MCC, and its advantage over the next-best model exceeded the corresponding fold-wise standard deviations. This suggests that the improvements in Table 4 are not due to random variation alone. More formal statistical comparisons (e.g., DeLong’s test for correlated AUCs^20^ or non-parametric tests recommended for classifier comparison^21^) could be applied in future multi-dataset studies, but are beyond the scope of this single-dataset industrial case study.

For comparison^5^, trained XGBoost on the Bosch data (80%/20% split) using only numeric features. They report AUC-ROC=0.997 and MCC=0.994. In contrast, our model (using WoE-compressed nominal features) achieved AUC-ROC=0.966 and MCC=0.793. As Nikolova et al. note, including all features (numeric, date, categorical) hurt performance, so they excluded categorical codes. Our approach differs by incorporating categorical risk via WoE encoding. We also note that Kaggle competitors on the Bosch challenge achieved much lower MCC scores; for example, the best private-leaderboard result was only about 0.487. This underscores how challenging the task is and explains why even strong models can show substantial metric differences.

The advantage of XGBoost in this evaluation is expected for three reasons. First, boosted trees capture non-linear interactions between station trajectories, timing aggregates, and sparse nominal risk indicators, which are typical in complex production paths. Second, XGBoost’s regularization and shrinkage reduce overfitting in wide tabular data, while its split/leaf weighting implicitly emphasizes “hard” minority failures during boosting rounds. Third, XGBoost handles missingness and heterogeneous feature scales naturally, which aligns with Bosch-style IIoT tables. Random Forest performs second-best because bagging stabilizes variance, but unlike boosting it does not concentrate sequentially on rare misclassified failures, so its MCC remains lower under extreme imbalance. Linear SVM and Factorized Machines lag because they model mainly linear or low-order interactions and cannot exploit the higher-order dependencies encoded by our station/path aggregation and WoE features.

Importantly, these gains are enabled by our feature-compression pipeline: the station/path numerical aggregates and WoE-based nominal descriptors expose higher-order interactions that simpler encoders or nominal-feature dropping would miss. This explains why XGBoost benefits most from the engineered representation, beyond its known general strength on tabular PdM tasks.

#### Multilayer Perceptron analysis

We trained multiple Multilayer Perceptron (MLP) models with varying numbers of hidden layers and neurons to explore different network architectures. The results of various Multilayer Perceptron architectures are presented in Table 5. The input layer always has a total of 193 neurons, corresponding to the final feature set after the preprocessing filter described in Section “Experimental setup”, while the output layer has two neurons, representing the two classes, one for defects and one for successful components. Optimal performance is achieved using two hidden layers, with one having 64 neurons and the other 32 neurons.

**Table 5:** Multilayer Perceptron model architecture and results.

**Model**	**Precision**	**Recall**	**F1-Score**	**AUC-ROC**	**MCC**
MP [193, 128, 2]	0.8505	0.9871	0.8431	0.8820	0.6011
MP [193, 64, 2]	0.8438	0.9788	0.8297	0.8442	0.5595
MP [193, 32, 2]	0.8541	0.9809	0.8447	0.8778	0.5987
MP [193, 128, 64, 32, 2]	0.8404	0.9721	0.8218	0.8324	0.5334
MP [193, 128, 64, 2]	0.8352	0.9634	0.8104	0.8005	0.4972
MP [193, 64, 32, 2]	**0.8550**	**0.9868**	**0.8489**	**0.8820**	**0.6147**

**Fig. 3:**
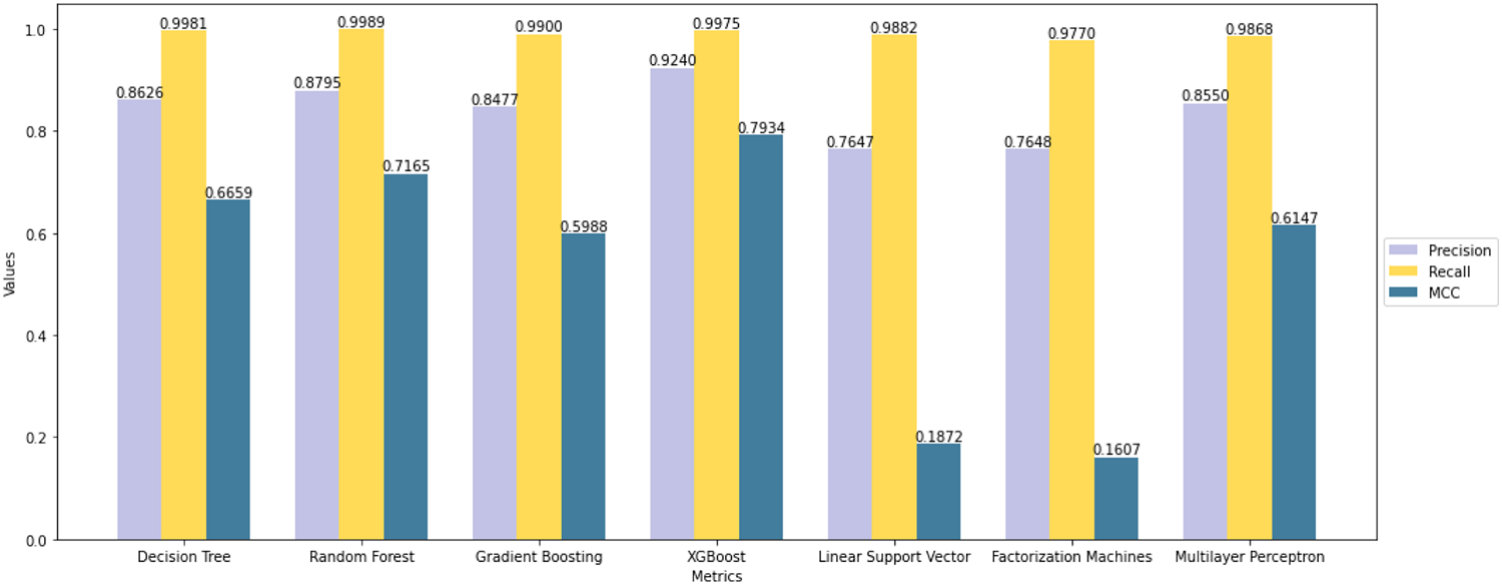
Comparison of Precision, Recall, and MCC across evaluated models on the test set under extreme imbalance (0.58% defects). XGBoost achieves the highest MCC, indicating best minority-class discrimination.

The values of MCC range between −1 and 1. For each of the models, the MCC value is positive, indicating that binary classification is relatively successful, with higher values suggesting better performance. Values above 0.6 indicate that despite the imbalanced classes, the model successfully distinguishes and classifies the minority class, as researched by Boughorbel, Jarray, and El-Anbari (2017)^22^. While each model exhibits high precision and recall, the difference lies in MCC, where each model has distinctive outlier values. This is visualized in Figure 3. where we observe variations in MCC values for each model, with XGBoost standing out for its superior performance.

#### Scalability and execution time

**Fig. 4:**
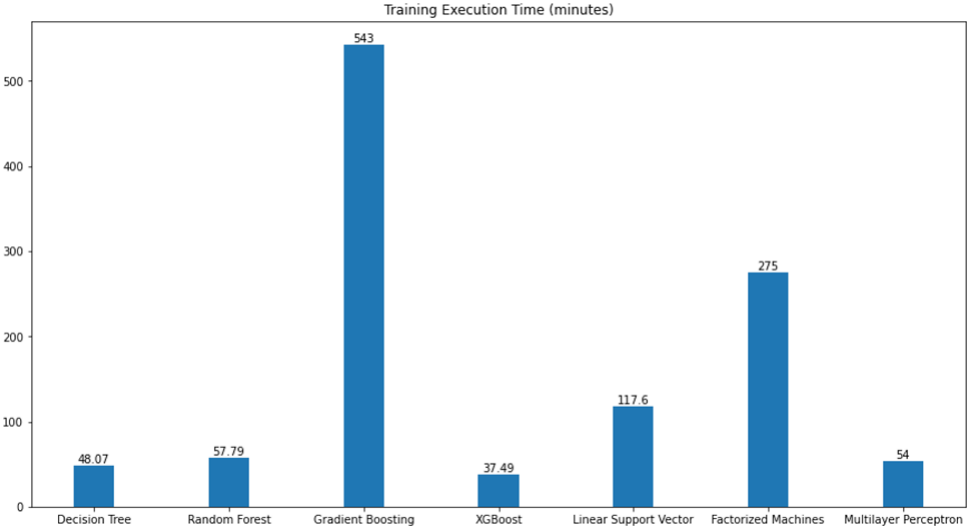
Model training time on the full Bosch dataset in Databricks/PySpark. XGBoost offers the best accuracy–runtime trade-off, supporting periodic retraining in Industry 4.0 environments.

In Figure 4, we have an overview of the training time for each model. Training time refers to the duration needed to build the machine learning model using historical data. The runtime is a critical factor in production, especially when predicting defects and ensuring the smooth operation of production lines. XGBoost not only excels in performance but also has the shortest training time. Faster model training enables quicker deployment for real-time predictions. A training time of 30 minutes for XGBoost is a favorable option, striking a balance between model accuracy and swift training, making it suitable for production environments. Once trained, however, the model’s inference on new parts is effectively real-time (milliseconds per prediction in our environment), which is the critical requirement for online monitoring. Moreover, once trained, the model’s inference on new data is nearly instantaneous (on the order of milliseconds per component), enabling true real-time defect prediction on streaming IIoT data. With the ability to make timely predictions for potential defects, manufacturers can take immediate actions, such as machine shutdowns, operator warnings, or initiating maintenance procedures. Faster model training not only improves response time to potential issues but also enhances cost efficiency by minimizing production downtime.

#### Misclassification analysis

To better understand the model’s behaviour, we inspected misclassified samples and typical failure cases from the best-performing XGBoost model. The few false negatives (missed defects) typically corresponded to parts whose aggregated station and WoE features were very close to those of non-defective parts, suggesting borderline conditions that are inherently difficult to separate. Most false positives, in contrast, involved components with unusual but ultimately non-failing patterns (e.g., atypical paths, extreme timing, or rare station-code combinations) that resembled failure signatures, leading the model to conservatively flag them as risky. These failure-case insights are important for industrial deployment because they indicate where additional process context or human-in-the-loop review may be beneficial, and they suggest that incorporating richer operational signals (e.g., station-specific constraints or temporal trends in streaming logs) could further improve reliability.

### Benefits from the engineered feature space

Our feature engineering step converts sparse, high-cardinality IIoT event identifiers (e.g., station/line/path codes) into a compact set of dense, ordered supervised statistics (WoE) and aligns them with aggregated numerical streams at the same operational granularity. Gradient-boosted trees (and XGBoost in particular) are well matched to this representation because each split is a simple threshold on one engineered scalar; the ensemble can then compose many such splits into a flexible piecewise-constant approximation that captures non-linear effects and cross-signal interactions without requiring explicit cross-features or one-hot expansions. In addition, boosting’s stage-wise error-correction tends to accumulate signal from many weak, sparse predictors – a common regime in production data where individual station indicators are only marginally informative but become predictive in combination. Finally, XGBoost’s regularization and feature subsampling help control overfitting when the minority class is extremely rare, while still supporting fast retraining on large tabular data. This behavior is consistent with applied predictive-maintenance deployments on manufacturing IoT streams, where ensemble trees (including XGBoost) have been reported among the best-performing models and integrated into operational systems^23^.

### Limitations

This study evaluates the approach on a single public industrial benchmark (Bosch Production Line Performance), where labels reflect final pass/fail outcomes rather than station-level fault timing. Whereas this makes the dataset suitable for defect prediction, it limits direct conclusions about root-cause localization or fully temporal condition-based maintenance.

In addition, the minority class is extremely rare (0.58%), so transferring the model to other factories, product families, or process setups may require recalibration due to distribution shifts. Given that the station-code distributions that will differ across factories, products, and measurement systems, transferring the approach to a new environment will require recalculating WoE mappings, monitoring distribution shift and applying drift-aware retraining.

Our current validation is based on offline training with capabilities of near real-time inference, so we do not yet report end-to-end performance in a live streaming deployment. Finally, while we report cross-validated variability and discuss standard statistical tools for classifier comparison, a full significance analysis across multiple independent datasets is left for future work.

For real IIoT event logs with temporal autocorrelation and concept drift, evaluation should use time-based splits and periodic retraining. However, this analysis is outside the scope of this study because the dataset lacks context about the exact industrial setup in which it was produced. Future studies should include a time-ordered sensitivity check to partially address this. One approach to achieve that is to add a time-ordered sensitivity analysis using a forward-chaining split (train on earlier parts, validate on later parts) based on the earliest available timestamp per part.

## Conclusion

This paper presents an approach for predicting manufacturing defects using the Bosch dataset, which tracks components across four production lines. The dataset presents challenges due to its size and the diverse range of features with various data types.

Selected features were used to train and evaluate various machine learning models. Parameter tuning significantly improved the performances of certain models, notably seen in Random Forest, where MCC increased from 0.4 to 0.7. Traditional models were compared against deep learning counterparts, and the training time was emphasized as a critical factor in production. Faster training is crucial not only for response time but also for cost efficiency by minimizing downtime. Our findings highlight the exceptional performance of the XGBoost model, demonstrating superiority in accuracy metrics, including 0.9660 AUC-ROC, 0.7934 MCC, and 0.9288 F1-score, as well as training time efficiency. A modern deep tabular baseline (TabNet) was also evaluated, achieving competitive performance but not surpassing XGBoost under this extreme-imbalance IIoT setting.

The proposed supervised defect prediction approach using machine learning models has significant implications for the manufacturing industry. Feature engineering overcomes challenges posed by IIoT data streams, and the use of Databricks provides resilience to such a system. Manufacturers can proactively prevent downtime, optimize maintenance schedules, and minimize production losses by precisely predicting defects. Consequently, the efficiency and productivity of manufacturing processes can be enhanced, leading to cost savings and increased profitability.

With this work, we align with the Industry 4.0 paradigm and provide an implementable blueprint for intelligent defect prediction in large-scale IIoT settings, while acknowledging that further validation on additional manufacturing environments is required before full generalization can be claimed.

Future work will need to validate the proposed pipeline in actual streaming environments (including latency, throughput, and resource usage), assess generalization across multiple manufacturing sites and product types, and conduct deeper failure-case analyses together with domain experts to better understand false alarms and missed defects.

## Data Availability

Data supporting the findings of this study are available at https://www.kaggle.com/c/bosch-production-line-performance/data.

## Code availability

The code used for the model training and evaluation is available at https://github.com/admin-magix/bosch-production-line-performance/.
